# Structure–property relationships in fibrous meniscal tissue through image-based augmentation

**DOI:** 10.1098/rsta.2024.0225

**Published:** 2025-03-13

**Authors:** Arash Rabbani, Ali Sadeghkhani, Andrew Holland, Mohsen Besharat, Han Fang, Masoud Babaei, Olga Barrera

**Affiliations:** ^1^ School of Computer Science, University of Leeds, Leeds, UK; ^2^ School of Civil Engineering, University of Leeds, Leeds, UK; ^3^ Department of Chemical Engineering, University of Manchester, Manchester, UK; ^4^ School of Engineering, Computing and Mathematics, Oxford Brooks University, Oxford, UK

**Keywords:** meniscus, image generation, biomaterial

## Abstract

This study introduces an adaptive three-dimensional (3D) image synthesis technique for creating variational realizations of fibrous meniscal tissue microstructures. The method allows controlled deviation from original geometries by modifying parameters such as porosity, pore size and specific surface area of image patches. The unbiased reconstructed samples matched the morphological and hydraulic properties of original tissues, with relative errors generally below 10%. Additional samples were generated with predefined deviations to increase dataset diversity. Analysis of 1500 synthesized geometries revealed relationships between microstructural features, hydraulic permeability and mechanical properties. Empirical correlations were derived to predict longitudinal and transverse hydraulic permeability as functions of porosity, with *R*^2^ values of 0.98 and 0.97, respectively. Finite-element simulations examined mechanical behaviour under compression, showing stress concentrations at fibre cross-links and permeability reductions that varied with porosity and flow direction. These results led to a porosity-dependent model for normalized Young’s modulus ($R^{2}=0.687$). The proposed correlations and data augmentation technique aid in investigating structure–property relationships in meniscal tissue, potentially benefiting biomimetic implant design. This approach may help bridge data gaps where obtaining numerous real samples is impractical or unethical.

This article is part of the theme issue ‘Uncertainty quantification for healthcare and biological systems (Part 1)’.

## 1. Introduction

The menisci, crescent-shaped fibrocartilaginous tissues located within the knee joint, play a crucial role in maintaining joint health and function. These structures act as natural hydraulic dampers, absorbing mechanical shocks and distributing load throughout the tibiofemoral joint [1,2]. The unique biomechanical properties of the meniscus are intrinsically linked to its complex microstructure, which consists of a highly organized network of collagen fibres, proteoglycans and other extracellular matrix components [3].

Understanding the complex relationship between the meniscus microstructure and its functional properties is essential for several reasons. It provides insights into the tissue’s normal physiology and how it responds to mechanical stresses. This knowledge is also crucial for diagnosing and treating meniscal injuries, which are among the most common knee-related problems [2]. Additionally, a deep understanding of meniscal microstructure is vital for developing effective tissue engineering approaches and biomimetic implants that can successfully replicate the function of native meniscal tissue [4].

Recent advances in imaging technologies have significantly enhanced our ability to analyse the microstructural organization of meniscal tissue. Techniques such as micro-computed tomography (μCT) and multiphoton microscopy have been employed to investigate the two-dimensional (2D) and three-dimensional (3D) architecture of meniscus samples in both healthy and pathological states [3,5]. For instance, Karjalainen *et al*. used μCT to compare the 3D collagen organization in meniscus samples from osteoarthritis (OA) patients and non-OA donors, revealing important insights into how alterations in collagen microstructure contribute to meniscus degradation in OA [5]. Using desktop μCT, [6] Kestilä *et al*. developed a high-resolution 3D imaging protocol for meniscus microstructures. Their analysis revealed that medial OA menisci had significantly higher histopathological scores compared with lateral OA and medial reference menisci, indicating a strong link between meniscus degradation and unicompartmental knee OA [6]. In addition, structural analysis using transmission electron microscopy has revealed significant differences in extracellular matrix organization, collagen fibre structure and cellular morphology between healthy, traumatically injured and OA menisci. Battistelli *et al.* [7] observed disorganization of collagen fibres, increased proteoglycan content and cellular degenerative changes in both traumatic tear and OA samples [7]. Furthermore, structural analysis via μCT has illuminated critical features such as porosity distribution, preferred flow pathways and flow tortuosity within different meniscus segments [8,9]. These factors are not only important for understanding the tissue’s native function but are also crucial considerations in the development of tissue engineering therapies [4,10]. For example, Agustoni *et al*. [8] revealed a network of collagen channels in the body region of the knee meniscus using high-resolution μCT, which has implications for both the tissue’s mechanical behaviour and its nutrient transport capabilities [8].

Previous studies have explored structure–property relationships in meniscal tissue using various experimental approaches. Kleinhans & Jackson investigated the anisotropic and inhomogeneous nature of meniscus permeability through direct permeation tests, finding that permeability was higher in the circumferential direction compared with the axial direction [11]. Morejon *et al*. examined compressive properties and hydraulic permeability of human meniscus samples, revealing correlations between mechanical parameters and tissue composition [12]. As another example, Berni *et al*. conducted a comprehensive assessment of both solid- and fluid-phase properties across different meniscal regions and loading directions, demonstrating regional variations in mechanical response and proposing a transversely isotropic model for the meniscus body [13]. These studies have provided valuable insights into how meniscus structure influences its functional properties but were limited by small sample sizes and the destructive nature of mechanical testing. These limitations stem from ethical considerations, the scarcity of donor tissues and the practical challenges associated with harvesting and preparing samples [14,15]. Consequently, there is a critical need for methods that can augment the available data and facilitate more extensive studies on structure–property relationships in meniscal tissue.

Image synthesis techniques offer a promising solution to overcome data scarcity issues in microstructure analysis. Data-driven techniques to tackle this problem include generative adversarial networks (GANs), exemplar-based methods and statistical approaches [16–18]. GANs use an adversarial process to generate realistic 3D microstructures from 2D or 3D images while subjected to extensive training and careful considerations to avoid overfitting [16,19–21]. Exemplar-based methods utilize existing microstructure images as templates to generate new, similar structures [22–24]. For instance, Kopf *et al*. developed a technique for synthesizing 3D solid textures from 2D exemplars [22]. This category also encompasses various in-painting techniques, as demonstrated by Criminisi *et al*. [25], Le Meur *et al*. [26] and Yang *et al*. [27] [25–27]. Statistical reconstruction methods, primarily rooted in geostatistics, employ multiple-point statistics for 3D reconstruction of porous media, as shown by Mariethoz & Renard [28].

However, most of the existing methods for 3D image synthesis and reconstruction focus on creating faithful reproductions of the original geometry textures [16,22,29]. Although this approach is valuable for certain applications, it limits the ability to explore a wider range of structural variations that might occur naturally or be desirable in engineered tissues. There is a need for adaptive techniques that can intentionally deviate from the original geometries while maintaining key structural characteristics, thus creating a more diversified range of reconstructions. We aim, in this study, to address this gap by proposing a 3D exemplar-based reconstruction technique.

## 2. Material and methods

### (a) Data description

The data used in this study are sourced from μCT scans of human meniscal tissue samples, as described by Waghorne *et al*. [9]. Specifically, we utilize three volumes of interest (VOI) extracted from the body region of a healthy medial human meniscus: Tissue #1 (VOI(1), 2 mm × 3 mm), Tissue #2 (VOI(2), 2 mm × 3 mm) and Tissue #3 (VOI(3), 1 mm × 3.13 mm). The samples were extracted from the central body of the meniscus using a surgical knife and immediately freeze-dried following the procedure detailed by Waghorne *et al*. [9]; μCT scans were performed using a Skyscan 1272 scanner (Bruker) with a resolution of 6.25 μm. The resulting image datasets consist of 1640 × 2452 pixel slices in the horizontal (*x–y*) plane. These images were processed using N-Recon software to eliminate artefacts and reduce noise in each slice and then thresholded to distinguish between void and solid elements [9]. A greyscale threshold value of 34 was used by Waghorne *et al*. [9] to binarize the images. Key architectural parameters such as porosity, channel diameter, connectivity and tortuosity were extracted from the image data as detailed in [9].

### (b) Geometry reconstruction

**Figure 1.**
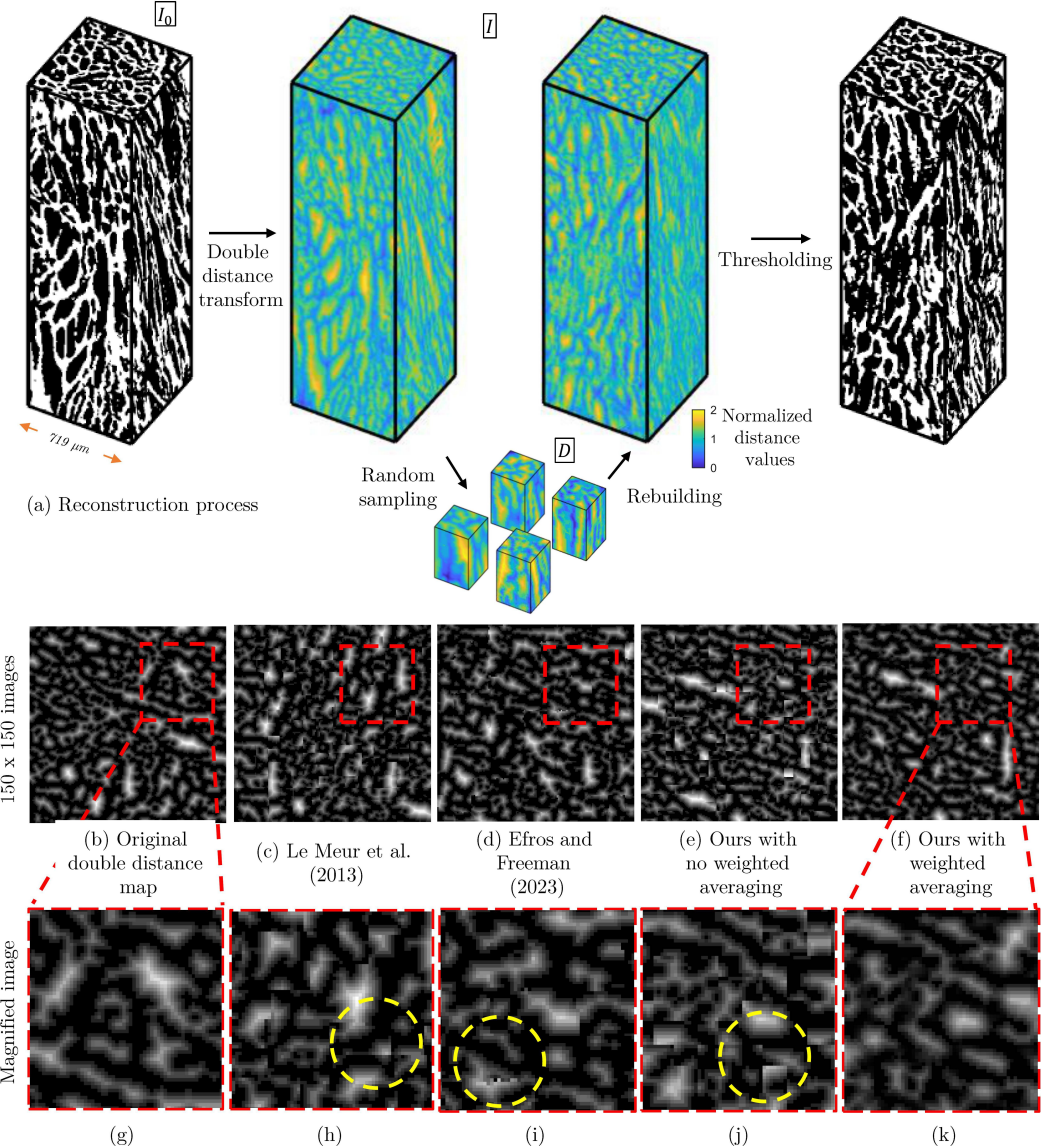
Schematics of the image reconstruction process, including breaking down the double distance map into small samples and rebuilding by putting them back together in a new order (*a*), a 2D slice of the double-distanced geometry (*b*), an examples of 2D reconstruction via Le Meur *et al.* [26] approach (*c*), an examples of 2D reconstruction via Efros & Freemain [30] approach (*d*), ours without weighted averaging (*e*) and ours with weighted averaging (*f*). A magnified region (*g–k*) shows detailed comparison of the reconstruction quality, with yellow dashed circles highlighting areas where discontinuities are visible in existing methods (*h–j*) but are effectively addressed by our weighted averaging approach (*k*).

The reconstruction process consists of three main phases: (i) preparation of a double distance map, (ii) creation of a diverse dataset through sampling and transformation and (iii) reconstruction of a new volume using this dataset and thresholding. The workflow for the reconstruction of geometries is illustrated in figure 1a. In this section, we discuss the three phases:

#### (i) Creating the double distance map

To generate new realizations of the 3D porous microstructures, we generalize a legacy 2D patch-based image synthesizing technique [23,24,31] to be suitable for tackling the high dimensionality of the volumetric images. Another variation is to adapt the method to work with binary porous material images. Considering the interpolation limits in the discretized space of binary images, we propose to convert it to a more spatially correlated grey-scale image which is in other words a double distance map (I) that transforms both the void and solid space of the image into a 3D field with intensities that show the normalized distance of each pixel from the interface between the two phases. A variation of this distance mapping has been presented by Rabbani *et al*. [32] to interpolate between two binary patterns. The double distance map (I) is defined as


(2.1) $$I=\hat{d}(I_{0})-\hat{d}(1-I_{0})+1,$$

where $I_{0}$ is the binary input image, in which the solid space is indicated by 1 and the pore space is denoted by 0, $\hat{d}(I_{0})$ is the normalized Euclidean distance transform of the binary image and the term $1-I_{0}$ inverts the binary image. The resulting normalized distance values follow a continuous scale from 0 to 2 (figure 1a), where values closer to 1 indicate proximity to the interface between void and solid phases.

#### (ii) Creating the dataset

Given an input double distance map $I\in R^{S_{1}\;\times \;S_{2}\;\times \;S_{3}}$, where $S_{1}$, $S_{2}$ and $S_{3}$ represent the dimensions of the input image in *x*, *y* and *z* directions, respectively, we create a diverse dataset through the following steps:

1. Define a set of geometric transformations g(I,i) where i∈{1,...,7}.

(2.2) $$\begin{array}{r} \begin{array}{r} \begin{array}{r} g(I,i)=\left\{ \begin{array}{ll} I & \text{if }i=1\\ R_{3}^{8}\times I & \text{if }i=2\\ R_{3}^{-8}\times I & \text{if }i=3\\ R_{2}^{8}\times open(I,SE) & \text{if }i=4\\ R_{2}^{-8}\times close(I,SE) & \text{if }i=5\\ R_{1}^{8}\times erosion(I,SE) & \text{if }i=6\\ R_{1}^{-8}\times dilation(I,SE) & \text{if }i=7, \end{array}\right. \end{array} \end{array} \end{array}$$

where SE is the morphological structuring element, which in this study is a small sphere with a diameter of three pixels to preserve the majority of the original features, $R_{1}^{8}$ denotes the rotation matrix for $8^{\circ }$ around the first dimension axis; $8^{\circ }$ is a small arbitrary angle that is selected to minimize the amount of cropped information post-rotation. In addition, to add more variations, the double distance maps are shifted by a random value between −0.1 and 0.1 units and randomly scaled by ±10% of the original distance values.

- For each transformed image, extract N cubic samples of size $s_{1}\;\times \;s_{2}\;\times \;s_{3}$ at random locations:

(2.3) $$D_{i,n}=g(I,i)(x_{n}:x_{n}+s_{1},\;y_{n}:y_{n}+s_{2},\;z_{n}:z_{n}+s_{3}),$$

where $\left(x_{n},y_{n},z_{n}\right)$ are randomly selected coordinates satisfying:


(2.4) $$1\leq x_{n}\leq S_{1}-s_{1},\;1\leq y_{n}\leq S_{2}-s_{2},\;1\leq z_{n}\leq S_{3}-s_{3}.$$

- Combine all samples into dataset $D\in R^{s_{1}\;\times \;s_{2}\;\times \;s_{3}\;\times \;(7N)}$ by concatenating along the fourth dimension

(2.5) $$D=\overset{7}{\underset{i=0}{||}}\;\overset{N}{\underset{n=1}{||}}D_{i,n}.$$

#### (iii) Volume reconstruction

Using the created dataset D, we reconstruct a new volume as follows:

1. Initialize output volume $B\in R^{\left(S_{1}+s_{1})\;\times \;(S_{2}+s_{2})\;\times \;(S_{3}+s_{3}\right)}$ with zeros and define overlap margin m (typically six voxels).
2. Define grid spacing Δ=s−2m and placement locations:

(2.6) $$L=\{(x,y,z)\;|\;x=m+n_{1}\Delta_{1},\;y=m+n_{2}\Delta_{2},\;z=m+n_{3}\Delta_{3}\},$$

where $n_{1},n_{2},n_{3}$ are non-negative integers such that the resulting coordinates remain within volume bounds.

- For each location (x,y,z)∈L in a randomized order:

- Extract current subsample and define occupancy mask

(2.7) $$b_{xyz}=B(x-m:x+s_{1}+m,\;y-m:y+s_{2}+m,\;z-m:z+s_{3}+m),$$

(2.8) $$\begin{array}{r} W_{xyz}=\left\{ \begin{array}{ll} 1 & \text{if }b_{xyz}\neq 0\\ 0 & \text{otherwise}. \end{array}\right. \end{array}$$

- Find optimal sample index $p_{best}$ from dataset D by minimizing matching error

(2.9) $$p_{best}=\underset{p\in \{1,...,7N\}}{arg\;min}\frac{\sum_{i,j,k}|b_{xyz}(i,j,k)-D_{p}(i,j,k)|\cdot W_{xyz}(i,j,k)}{\sum_{i,j,k}W_{xyz}(i,j,k)},$$

where $D_{p}$ represents the p-th sample in dataset D and the error is computed only in the overlapping regions where $W_{xyz}=1$. The summation over (i,j,k) represents the element-wise operations across all voxels in the sampling window.

- Calculate blending weights using distance transform

(2.10) $$w_{xyz}=\frac{d(W_{xyz})}{\max(d(W_{xyz}))},$$

where d(⋅) computes the Euclidean distance to nearest non-zero voxel.

- Update output volume with weighted averaging

(2.11) $$B(x:x+s_{1},\;y:y+s_{2},\;z:z+s_{3})=b_{xyz}\cdot w_{xyz}+D_{p_{best}}\cdot (1-w_{xyz}).$$

This process continues until all blocks in the output volume are filled with proper patches.

- Final output is obtained by cropping, smoothing and thresholding

(2.12) $$B_{final}=(G_{\sigma }(B(1:S_{1},\;1:S_{2},\;1:S_{3}))-1)>0,$$

where $G_{\sigma }$ is Gaussian smoothing with standard deviation σ, typically as small as 0.5 to exclude minor discontinuities between adjacent patches while preserving the texture. The shifting of 1 unit is to revert the value shift in equation (2.1), and the logical operation > 0 inherently converts the result to a binary image.

It is noteworthy that as the reconstruction progresses, finding matching edges for remaining blocks becomes increasingly challenging owing to the growing number of boundary conditions imposed by previously placed blocks. Consequently, the final blocks may have fewer matching edges with neighbours, potentially creating discontinuities in the microstructure. To address potential discontinuities, as shown in equation (2.11), we implement a weighted averaging technique between existing and newly placed pixels, resulting in smoother transitions. The interpolation weight gradually decreases as we move away from block borders, allowing the original pixels to become more prominent. Figure 1b–f illustrates the effect of weighted averaging on reconstruction quality, with a magnified region (figure 1g–k) providing a detailed comparison. Both Le Meur *et al*. ([26]; figure 1c,h and Efros and Freeman ([30]; figure 1d,i, as well as our method without weighted averaging (figure 1e,j) exhibit discontinuous patterns, as highlighted by the yellow dashed circles. In contrast, figure 1f,k demonstrates a smoother transition between image blocks, achieved through our proposed weighted averaging technique. For a more comprehensive explanation of the reconstruction details, please refer to the 2D implementation described by Rabbani *et al*. [24]. As a note, they have also attempted to measure the diversity of their generated 2D dataset by comparing the area covered in the porosity versus specific-surface feature space. They observed a five-times increase in the area covered in feature space compared with the original geometry.

**Figure 2.**
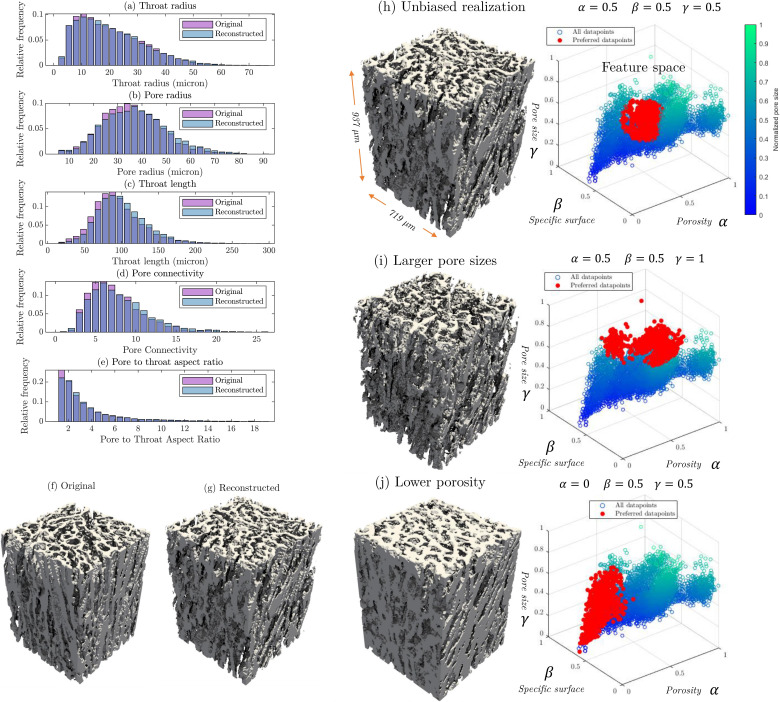
Comparison of pore network statistics for the original and an unbiased reconstruction of the of tissue sample #1 (*a–e*), 3D rendering of the both geometries (*f,g*), unbiased (*h*), large pore (*i*) and low-porosity (*j*) realizations of tissue sample #1 and their preferred data points in feature space of α (normalized porosity), β (specific surface area) and γ (maximum pore size).

The method described in this subsection is designed to reconstruct porous structures that closely resemble the original in appearance, morphological properties (figure 2a–g) and hydraulic characteristics (to be discussed later). However, to intentionally create variations from the original structure, we can impose conditions that guide the model to generate realizations with similar texture but altered porosity, specific surface area and pore size. To achieve this selective behaviour, we extract these three key features from the four-dimensional dataset D and use them to filter out entries that deviate significantly from the desired range. We quantify feature distances by defining a 3D conceptual space with axes (α, β, γ) representing normalized porosity, specific surface area and pore sizes, respectively. Each axis ranges from 0 to 1. To generate new realizations, we select 1/8 of the data points closest to the desired settings in this feature space. For example, setting α=0.5, β=0.5, γ=0.5 retains the 1/8 of entries in the dataset for which features are closest to this target point. This creates a sphere in the feature space, resulting in an unbiased realization that closely matches the original geometry (figure 2h). Conversely, setting α=0.5, β=0.5, γ=1 reconstructs a geometry with relatively large pores, potentially sacrificing some similarity in specific surface area or porosity (figure 2j). Alternatively, setting α=0 reduces the porosity of the generated sample (figure 2i) down to its practically possible minimum. This adaptive reconstruction approach allows us to synthetically fill data gaps that exist owing to practical or ethical limitations in acquiring real tissue samples. Consequently, we can conduct regression studies to investigate the relationship between the properties of the studied tissues and their morphology.

### (c) Hydro-mechanical analysis

To investigate structure–property relationships in the reconstructed geometries, we employed three analysis approaches, with full details provided in appendices A–C. The hydraulic properties were analysed using a pore network approach based on marker-based watershed segmentation ([33–36]; appendix A). This method segments the pore space into distinct regions and models fluid flow through the resulting network, enabling calculation of both longitudinal and transverse permeabilities.

The mechanical behaviour was examined through finite-element simulations using FEBio [37] with neo-Hookean material modelling ([38–43]; appendix B). Samples were subjected to 15% compression, consistent with previous studies [44], while analysing stress distributions and displacement patterns in relation to the tissue’s fibrous structure [2,45].

For larger-scale tissue analysis, we developed empirical correlations between structural and physical properties through regression analysis of 1500 generated samples. The methodology for mapping CT numbers to porosity values, based on approaches similar to those used in bone density studies [46,47], is detailed in appendix C. This mapping enabled the extension of our structure–property relationships to whole-tissue scales.

## 3. Results and discussions

This section examines the reconstructed meniscal tissue geometries and their properties. Initially, the physical properties of synthesized samples are compared with those of the original tissues to validate the reconstruction method. The adaptive reconstruction approach then generates diverse tissue geometries, enabling exploration of relationships between tissue structure and function. Particular focus is given to the influence of porosity on hydraulic permeability and elasticity. Finite-element simulations reveal mechanical behaviour under compression, including stress distributions and permeability changes. Finally, the application of these findings to larger scale tissue analysis is demonstrated.

### (a) Morphology and hydraulic behaviour

Pore network modelling is performed on sample #1 and its unbiased realizations. Table 1 lists a comparison between the morphological and physical properties of the original tissue sample #1 and the average properties of 30 reconstructed images (with α=0.5, β=0.5 and γ=0.5). The properties examined include pore radius, throat radius, pore connectivity (number of pores connected to each pore), porosity, specific surface area and hydraulic permeability. For all these properties, the relative error between the original and reconstructed samples is less than 10%.

**Table 1.** Physical properties of the original tissue sample #1 and their average for 30 reconstructed samples as well as the relative error of the average properties.

properties	original	reconst.	error (%)
pore radius (µ)	34.47	36.16	4.9
throat radius (µ)	18.94	19.42	2.53
pore connectivity	6.5	7.03	8.15
porosity (fraction)	0.61	0.62	1.64
specific surface (1/mm)	87.62	87.2	0.48
hydraulic permeability (Darcy)	14.97	16.47	10.02

The reconstructed geometries were generated by repeating the process 30 times with different random seeds to obtain average values. Among all properties, hydraulic permeability shows the highest deviation, which is expected owing to its quadratic relationship with linear changes in throat size [35]. For the unbiased reconstructions, quantitative analysis of the matching quality shows that on average 93% of the blocks have a relative matching error less than 0.2 at their borders with neighbouring blocks, where the matching error is computed using the objective function described in equation (2.9). As discussed, figure 2a–e illustrate the distribution of these properties for the original sample and one of the reconstructed unbiased realizations (figure 2g). For the property distributions shown in figure 2a–e, an average relative error of 4.4% was observed in the relative frequencies.

**Figure 3.**
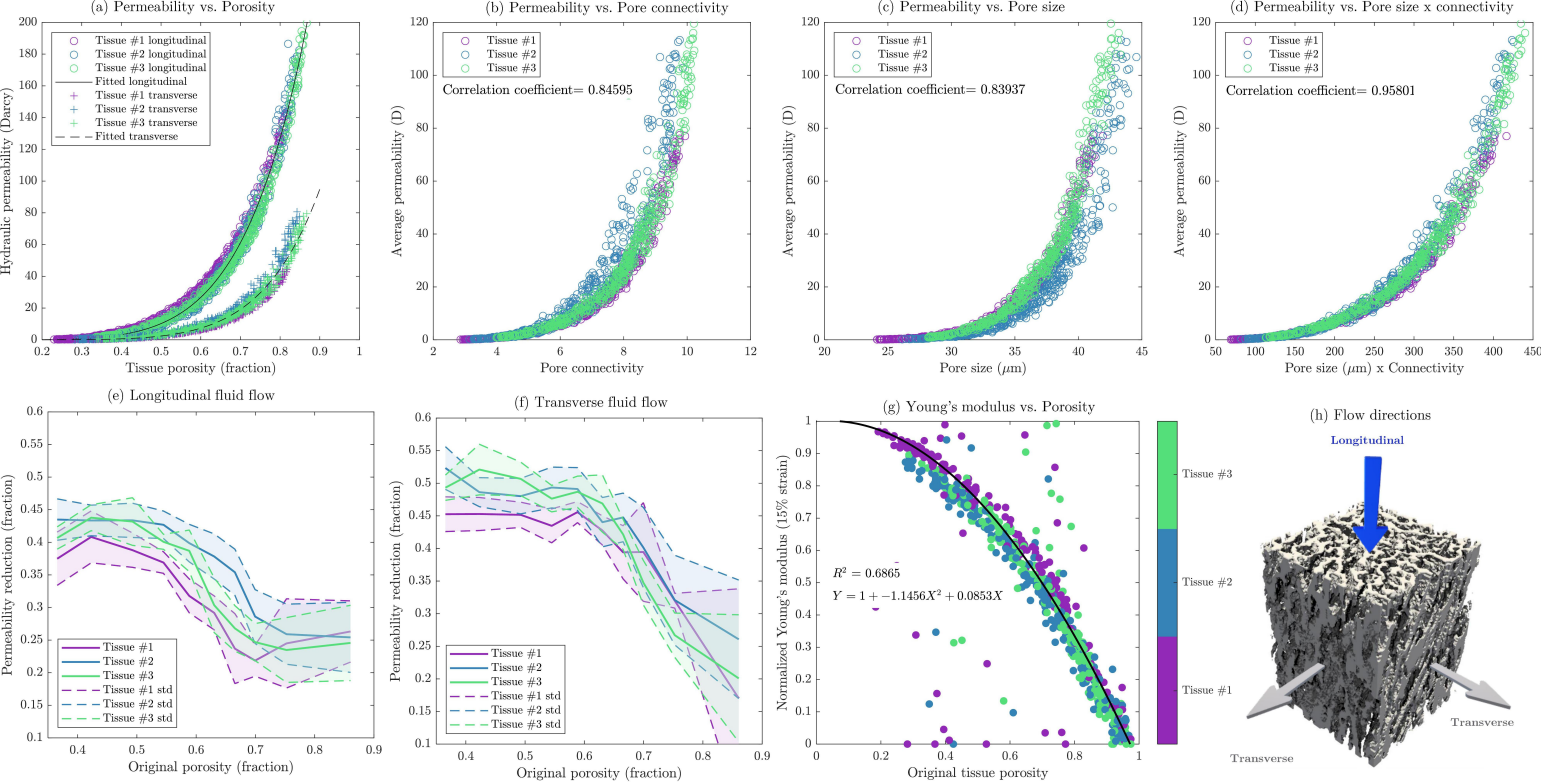
Relationship between the tissue hydraulic permeability (longitudinal and transverse) and porosity for three studied real examples that were used as the basis for reconstruction of 1500 realizations in total (*a*), effects of pore size (*b*) and pore connectivity (*c*) (coordination number) and the combined effect of both (*d*) in average hydraulic permeability of tissues, permeability reduction owing to 15% vertical strain for longitudinal (*e*) and transverse (*f*) fluid flow, changes in normalized Young's modulus versus original porosity of the tissue samples (*g*) and flow directions in relation to the sample orientation (*h*).


To demonstrate the application of the presented adaptive reconstruction approach in structure-property modelling, 1500 geometries were generated based on the described real μCT images with uniformly random sets of α, β and γ. The longitudinal ($K_{L}$) and transverse ($K_{T}$) hydraulic permeabilities of these geometries were compared as porosity changes. Figure 3a reveals a visible trend that can be modelled via empirical correlations. Several functional forms were tested to model this behaviour, including: (i) a cubic logarithmic exponential model with cubic terms in the exponent, (ii) a complete third-degree polynomial with all terms ($\phi^{3}$, $\phi^{2}$, ϕ) and (iii) a reduced third-degree polynomial with only cubic and linear terms ($\phi^{3}$, ϕ). After evaluating their performance through coefficient of determination ($R^{2}$) via cross-validation with fivefold, the following logarithmic exponential form was selected as it achieved the highest $R^{2}$ while maintaining model parsimony


(3.1) $$K_{L}=\exp(4.719\cdot \log(\phi )+\phi +5.097),$$

(3.2) $$K_{T}=\exp(5.567\cdot \log(\phi )+\phi +4.239),$$

where ϕ represents the porosity fraction and permeability is expressed in Darcy units. The determination coefficients ($R^{2}$) for the fitted equations are 0.98 and 0.97 for the longitudinal ($K_{L}$) and transverse ($K_{T}$) hydraulic permeability, respectively. The root-mean-squared errors of the equations are 5.5 and 2.1 Darcy, respectively.

The higher root-mean-squared errors observed in the longitudinal permeability are consistent with its higher average values, which are approximately twice the transverse permeabilities owing to the longitudinal fibre alignment. Given that porosity measurements are more readily accessible compared with hydraulic permeability, these equations offer potential for simplifying tissue characterization.

To further explore the structure–property relationships, the correlation between average hydraulic permeability and key microstructural parameters was examined (figure 3b–d). In this analysis, the permeability values used were arithmetically averaged over the three flow directions, representing an average flowability independent of the orientation of the fibre strands. The results reveal strong positive correlations, measured by the Pearson correlation coefficient (ρ), between permeability and both average pore connectivity (ρ = 0.8459, figure 3b) and average pore size (ρ = 0.8393, figure 3c). These findings align with effective medium theory approaches to porous media flow [48], which consider the effects of pore size distribution and network connectivity on overall permeability. Notably, an even stronger correlation (ρ = 0.9580, figure 3d) was observed between permeability and the product of pore size and connectivity. The improved predictive power of this combined metric highlights the interdependence of pore geometry and topology in determining hydraulic properties. Similar relationships between pore characteristics and fluid transport have been observed in other biological tissues, such as bone [49] and cartilage [50]. This finding has potential implications for tissue engineering approaches to meniscus repair or replacement. It suggests that optimizing both pore size and connectivity may be more effective in replicating the native tissue’s hydraulic properties than focusing on either parameter alone.

### (b) Tissue mechanical behaviour

**Figure 4.**
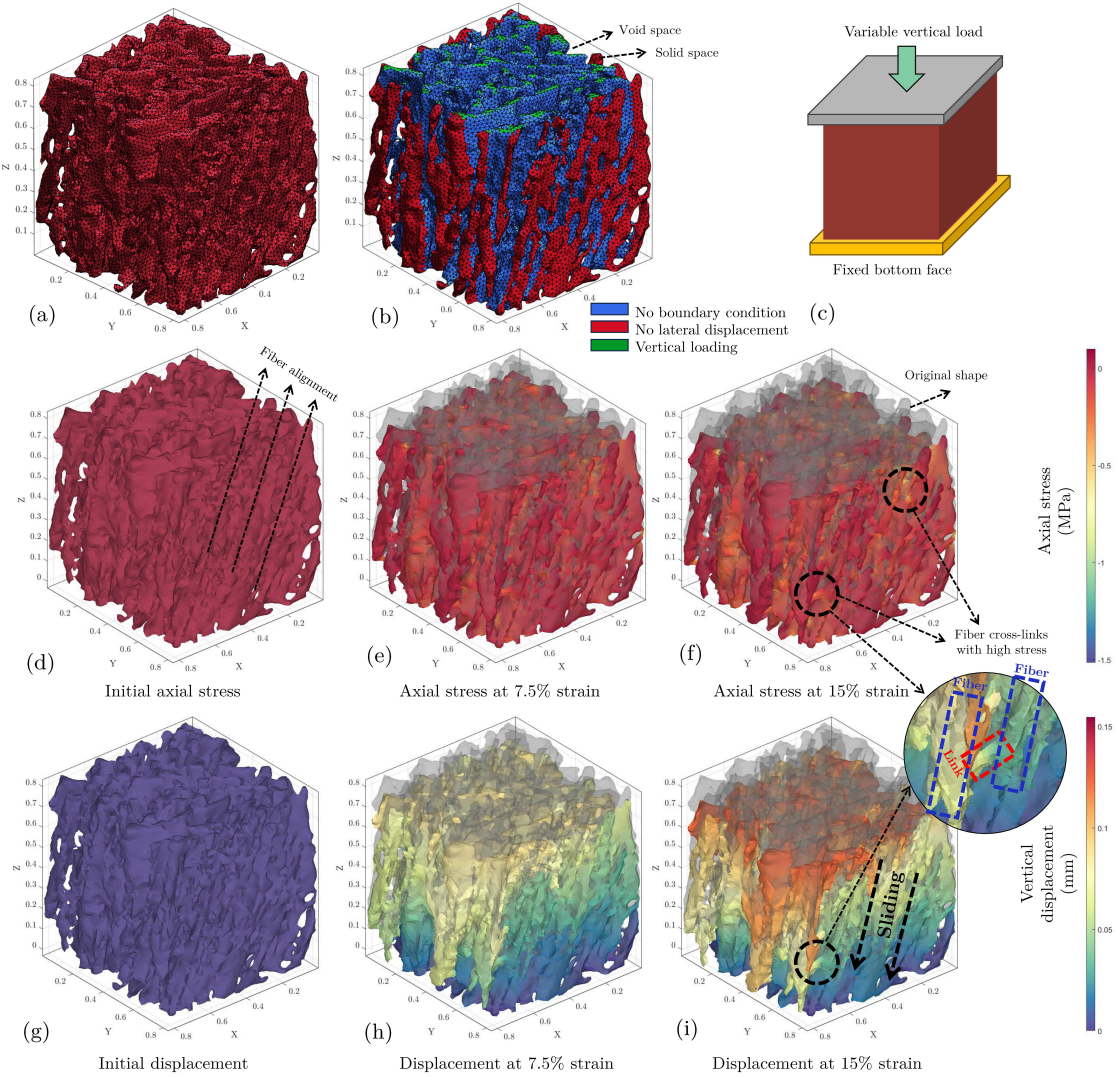
Finite-element compression modelling of a sub-image from tissue 1, (*a*) original mesh, (*b*) boundary conditions, (*c*) compression system schematic, (*d–f*) axial stress versus strain, (*g–i*) vertical displacementversus strain.

The mechanical properties of meniscal tissue were investigated using finite-element simulations on reconstructed geometries. From each of the 1500 realizations, a representative $110^{3}$ voxel sub-image at a resolution of 7.6μm per voxel was cropped and converted into a tetrahedral mesh using the iso2mesh package [51]. For the sub-sample from the original tissue #1, the resulting mesh contained approximately 23 000 faces and 12 000 nodes (figure 4a). These numbers could vary based on the porosity and complexity of the sample. Boundary conditions were applied to simulate confined compression, with the bottom surface fixed, 15% compression prescribed on the top surface and lateral expansion constrained (figure 4b,c). This compression level falls within the physiological range observed in *in vivo* studies and has been used in previous biomechanical analyses of meniscal tissue such as the work published by Son *et al*. [44] which tested the tissues for 5, 10, 15 and 20% of offset for compression tests.

The stress distribution during compression reveals higher stresses at fibre cross-link locations (figure 4d–f). This heterogeneous stress pattern is consistent with the complex microstructure of meniscal tissue and corroborates observations in previous studies [52]. The initial stress state (figure 4d) shows an inclined alignment of fibre strands, reflecting the tissue’s anisotropic nature [53]. Displacement patterns align with fibre orientation (figure 4h) and indicate fibre layers sliding over each other (figure 4i). This behaviour explains the high stress concentrations at cross-links.

In addition, the effect of compression on hydraulic permeability was examined for all reconstructions (figure 3e,f). The stress-induced permeability reduction ratio is defined as $(K_{start}-K_{end})/K_{start}$ which was calculated for both longitudinal and transverse fluid flow as a function of original porosity. Rendering of the flow directions in relation to the sample orientation is shown in figure 3h.

Higher porosity samples generally exhibited a more severe reduction in permeability, possibly owing to compromised structural integrity that allows more fibres to collapse. Longitudinal permeability reduction showed less sensitivity to porosity (figure 3e), probably because aligned fibres maintain some inter-strand gaps for fluid flow even under compression. Transverse flow in high porosity samples (75%) showed greater variability in permeability reduction (figure 3f), possibly owing to increased freedom for fibre strands to collapse and constrict lateral flow paths. Based on the mechanical simulation results, the relationship between normalized Young’s modulus and original porosity for the three tissue samples was also investigated and visualized in figure 3g. A quadratic fit yielded the equation


(3.3) $$E_{n}=1-1.1456\phi^{2}+0.0853\phi ,$$

where $E_{n}$ is the normalized Young’s modulus and ϕ is the porosity. This relationship, with an $R^{2}$ of 0.687, ensures a normalized modulus of 1 at zero porosity and approaches zero for high porosities. The equation reflects the loss of stiffness in sparse fibre arrangements. Partially similar trends between the porosity and compressive Young’s modulus along the collagen fibres direction were published by Granke *et al*. [54].

**Figure 5.**
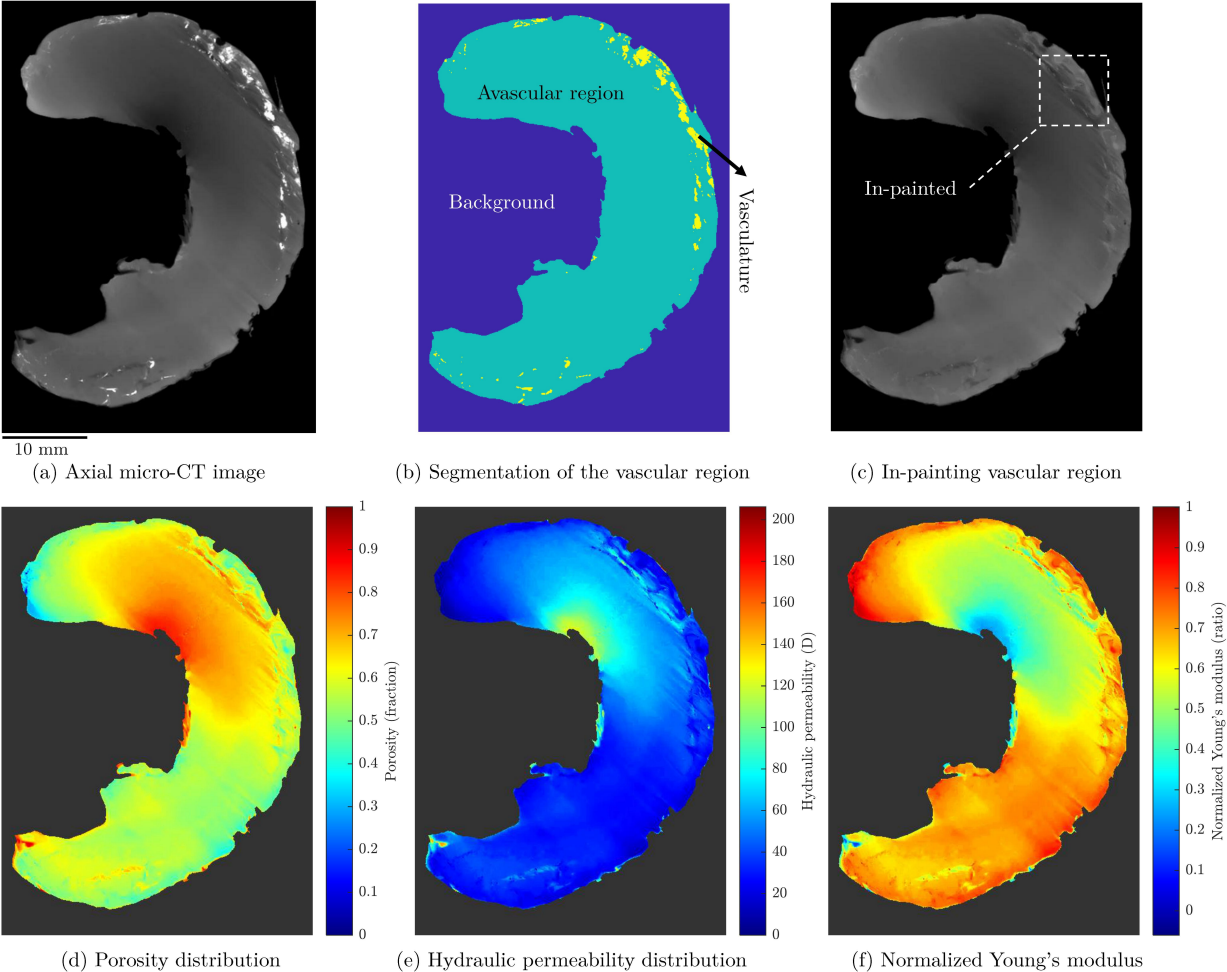
Conceptual inference of meniscus hydro-mechanical properties based on the μCT image assuming direct linear correlation of CT number with porosity, (*a*) Raw image adopted from [55] and slightly edited to exclude annotations, (*b*) segmentation of the vascular and avascular regions, (*c*) in-painting the vascular region, (*d*) porosity distribution map, (*e*) distribution of the hydraulic permeability based on equation (3.2), (*f*) distribution of the normalized Young's modulus based on equation (3.3).

To demonstrate potential applications in tissue characterization and engineering, we applied our structure–property relationships to analyse a larger scale meniscal tissue CT image (figure 5). Using the CT number to porosity mapping described by equation (C 3) (appendix C),we generated a detailed porosity distribution map of the tissue (figure 5d). The vascular region was first excluded and treated with coherent in-painting [56] to focus on the avascular region’s properties (figure 5a–c). The resulting porosity map revealed lower porosity at the outer edges of the meniscus, which aligns with findings from previous studies on meniscus microstructure [52] (figure 5d).

Based on this porosity distribution, we then generated maps of hydraulic permeability using equation (3.2) (figure 5e) and normalized Young’s modulus using equation (3.3) (figure 5f). While this approach relies on simplifying assumptions, it illustrates how microstructural variations may influence the tissue’s mechanical and hydraulic properties across larger scales.

Such mapping could potentially aid in understanding tissue function and pathology, similar to the work by [5] that mapped collagen fibre orientation between OA and healthy donor menisci. Furthermore, this method could provide valuable insights for tissue engineering applications, where understanding the spatial distribution of mechanical and hydraulic properties is crucial for designing biomimetic scaffolds or assessing the progress of tissue regeneration.

## 4. Conclusions

In the present study, a new adaptive 3D image synthesis technique is introduced to create variational realizations of fibrous meniscal tissues. The results of this exemplar-based method not only mimic the original image texture but also deviate from the original geometry by controlling parameters such as porosity, pore size and specific surface area. Taking into account the practical limits in obtaining a large number of tissues for regression studies on structure–property relationships, the presented data augmentation technique has the potential to bridge the data gaps and contribute to the characterization of biomimetic fibrous tissues. Key findings and outcomes of the present study include

— The unbiased 3D reconstruction method produced samples that closely matched morphological and hydraulic properties of original tissues, with relative errors generally below 10%.
— Analysis of 1500 augmented geometries revealed relationships between microstructural features, hydraulic permeability and mechanical properties:
  - Empirical correlations were derived for longitudinal and transverse hydraulic permeability as functions of porosity ($R^{2}$ = 0.98 and 0.97, respectively).
  - A strong correlation (R = 0.9580) was found between permeability and the product of pore size and connectivity.
  - A porosity-dependent model for normalized Young’s modulus was developed ($R^{2}$ = 0.687).
— Finite-element simulations of tissue compression showed:
  - Stress concentrations at fibre crosslinks
  - Displacement patterns aligned with fibre orientation
  - Permeability reductions that varied with porosity and flow direction.
— The technique enabled mapping of hydraulic and mechanical properties across larger scale meniscus images based on intensity values in CT images.

## 5. Limitations and future directions

This study, while innovative, has limitations. The reconstruction method relies on a limited sample set, potentially affecting result generalizability. In addition, our focus on static properties overlooks important time-dependent behaviours, and the assumed linear relationship between CT intensity and tissue properties requires further validation.

Future work should address these limitations and expand the research scope. Incorporating a larger, more diverse set of tissue samples would improve method robustness. Investigation of time-dependent fluid–solid interactions, including viscoelasticity and poroelasticity, would provide a more comprehensive understanding of tissue mechanics. Developing advanced techniques for mapping imaging data to tissue properties is crucial for improving the accuracy of large-scale tissue analysis. Also, integration of machine learning could enhance both reconstruction and property prediction processes. Extending the analysis to include additional mechanical properties such as tensile strength and shear modulus would provide a more complete characterization of tissue behaviour.

## Figures and Tables

**Figure 6.**
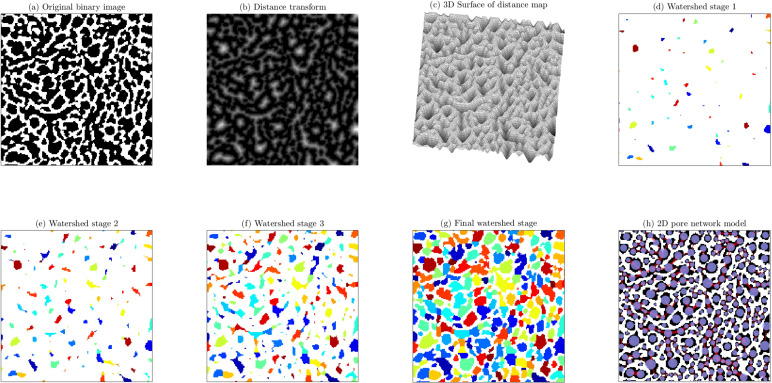
Step-by-step visualization of the watershed-based pore network extraction process in a 2D example: (a) Original binary image of the porous structure where black represents void phase and white represents solid space, (b) Distance transform map where brighter intensities indicate greater distances from solid boundaries, (c) 3D surface representation of the distance transform showing the topographical nature of the pore space, (d–g) Progressive stages of the watershed segmentation where different colours represent distinct pore regions growing from local maxima of the distance map, and (h) Final 2D pore network model overlaid on the original image, where detected pores are shown in different colours and their connections form the network structure.

## Data Availability

The developed image synthesis code is available at [57]. This research study was conducted retrospectively using data analyzed in [9] and STL files available at [58]. Supplementary material is available online [59].

## Appendix A. Hydraulic modelling details

The hydraulic properties of the meniscal tissue are modelled using a pore network approach based on marker-based watershed segmentation [33,34]. The watershed algorithm, originally inspired by geographical water basin formation, segments the pore space into distinct regions through a progressive flooding process.

The watershed segmentation process, illustrated step-by-step in figure 6, begins with a binary image of the tissue (figure 6a). This is transformed into a distance map (figure 6b), where each void pixel’s value represents its distance to the nearest solid boundary. This distance map can be visualized as a topographical surface (figure 6c) where peaks represent pore centres. The watershed algorithm then progressively segments the pore space (figure 6d–g) by simulating flooding from local minima of the inverted distance map. As the water level rises, boundaries (watersheds) form where different flood regions meet, defining individual pores. These boundaries delineate the pore space into distinct volumetric regions, each representing a single pore, ultimately resulting in a network model (figure 6h) where each colour represents a distinct pore.

Mathematically, this process begins with the segmentation of the 3D binary image $I_{0}$ of the tissue. The smoothed distance transform is computed and the watershed algorithm is applied:

(A 1)
$$L=W(-G_{\sigma }(\hat{d}(1-I_{0}))),$$

where $G_{\sigma }$ is a 3D Gaussian filter with standard deviation σ, W is the watershed segmentation function and L is the labelled image representing individual pores.

From this segmentation, a pore network model is extracted where each pore becomes a node in the network, and connections between adjacent pores form the network edges. For each pore i, its centroid $c_{i}$ and equivalent radius $r_{i}=(3V_{i}/4\pi {)}^{1/3}$ are calculated, where $V_{i}$ is the pore volume. The connectivity between pores is determined by analysing the interfaces between adjacent watershed basins, yielding throat radii $r_{ij}$ and lengths $l_{ij}$ for each pore-pore connection. The hydraulic conductance of each throat is calculated using an image-based throat permeability model [35], combined with the throat conductivity definition:

(A 2)
$$g_{ij}=\frac{k_{ij}A_{ij}}{l_{ij}}=\frac{\pi r_{ij}^{2}}{8\mu \|c_{i}-cj\|}(1.342D_{ij}^{2}-0.913D_{ij}-0.381),$$

where $D_{ij}$ is the average distance values of the throat cross-sectional image, μ is the fluid viscosity, $k_{ij}$ is the throat permeability, and $A_{ij}$ is the throat cross-sectional area.

To calculate the absolute permeability of the tissue, the pressure field p in the pore network is solved. A system of linear equations based on mass conservation at each pore i is constructed [36]:

(A 3)
$$\sum_{j\in N_{i}}g_{ij}(P_{i}-P_{j})=q_{i},$$

where $N_{i}$ is the set of pores connected to pore i, and $q_{i}$ is a source/sink term (non-zero only for inlet/outlet pores). This system can be expressed in matrix form AP=q, where A is a sparse conductance matrix. Dirichlet boundary conditions are applied to inlet and outlet pores, and the linear system is advised to be solved using the biconjugate gradient Stabilized method with an incomplete LU preconditioner for better computational efficiency.

Once the pressure field is obtained, the total volumetric flow rate Q across the sample is calculated by summing the flow rates through all inlet connections. The absolute permeability K is then derived using the generalized Darcy’s law at the macro-scale:

(A 4)
$$Q=\sum_{i\in \text{inlet}}\sum_{j\in N_{i}}g_{ij}(p_{i}-p_{j}),\;K_{t}=-\frac{\mu Q}{A_{t}}\frac{L_{t}}{\Delta P_{t}},$$

where $L_{t}$ is the total length of the sample in the flow direction, $A_{t}$ is the total cross-sectional area perpendicular to the flow direction and $\Delta P_{t}$ is the applied pressure difference. While the local pore-level flow behaviour is governed by the complex network of interconnected channels, this macro-scale permeability provides a useful metric for comparing different tissue samples and reconstructions. It is noteworthy that in steady-state condition with the laminar flow assumption, the applied pressure difference and fluid viscosity can be set arbitrarily and have no effect on the absolute hydraulic permeability $K_{t}$. However, future studies may benefit from incorporating time-dependent models to better capture the transient hydraulic response of the tissue under dynamic loading conditions.

## Appendix B. Mechanical modelling details

The mechanical behaviour of the meniscal tissue was modelled using a finite-element approach. To complement the hydraulic analysis presented in the paper and demonstrate another potential application of the augmented dataset, we employed a simplified quasi-static mechanical model focusing on basic structure-property relationships rather than fully coupled poroelastic behaviour. First, the 3D binary image of the tissue was converted into a tetrahedral mesh using the iso2mesh package [51] in MATLAB. The meshing process starts with finding normal vectors to the surface of the binary volumetric image, followed by the generation of a surface mesh and subsequent volumetric meshing. The mesh quality was controlled by setting a desired edge length of approximately 3.5 times the voxel resolution. The finite-element analysis was performed using FEBio [37], an open-source software for biomechanical simulations, with the model set-up and execution managed through the GibbonCode toolbox [60] in MATLAB. The meniscal tissue was modelled as a neo-Hookean hyperelastic material [38]. Considering the wide range of material properties reported in the literature for meniscal tissue [2,45], we focused on normalized compressive elasticity to enable comparison across different tissue samples and reconstructions. For this purpose, considering that our mesh represents the solid fibrous matrix of the meniscus, we modelled it as a slightly compressible material with a reference Young’s modulus of 1 MPa and a Poisson’s ratio of 0.25. This choice of compressibility is justified by experimental observations that meniscal tissue exhibits some volume change under compression [45], and the selected Poisson’s ratio falls within the range reported in literature for meniscal tissue [2]. Although individual collagen fibres are reported to exhibit Young’s moduli ranging from several MPa to a few GPa [61,62], we assign a reference modulus of 1 MPa to the fibre material for normalization purposes. This allows us to study the relative mechanical response of different network configurations independent of the absolute material properties. The network’s bulk behaviour emerges from the geometric arrangement of these fibres, which can undergo reorganization and collective deformation under loading, even before considering full poroelastic effects. In FEBio, the neo-Hookean material model is implemented using the strain energy function [39,40]:

(B 1)
$$W=\frac{\mu }{2}(I_{1}-3)-\mu \ln J+\frac{\lambda }{2}(\ln J{)}^{2},$$

where μ and λ are material coefficients known as Lamé parameters, $I_{1}$ is the first invariant of the right Cauchy–Green deformation tensor (representing the sum of squares of principal stretches in the material) and J is the determinant of the deformation gradient representing volume change. The Lamé parameters are related to the Young’s modulus E and Poisson’s ratio ν by [39]:

(B 2)
$$\mu =\frac{E}{2(1+\nu )},\;\lambda =\frac{E\nu }{\left(1+\nu )(1-2\nu \right)}.$$

The primary governing equation solved via FEBio is based on the conservation of linear momentum under quasi-static conditions [41]:

(B 3)
∇⋅σ=0in Ω,

where σ is the Cauchy stress tensor and Ω represents the solid portion of the tissue domain which is forming the computational mesh. The Cauchy stress tensor σ is derived from the strain energy function as [39,42,43]:

(B 4)
$$\sigma =\frac{1}{J}\frac{\partial W}{\partial F}F^{T}=\frac{\mu }{J}(B-I)+\frac{\lambda }{J}\ln(J)I,$$

where F is the deformation gradient tensor and $B=FF^{T}$ is the left Cauchy-Green deformation tensor [42]. The boundary value problem is defined with the following conditions:

(B 5)
$$\begin{array}{llll} u & =0 & & \;\text{on }\Gamma_{\text{bottom}}, \end{array}$$

(B 6)
$$\begin{array}{llll} u_{z} & =-0.15h & & \;\text{on }\Gamma_{\text{top}}, \end{array}$$

(B 7)
$$\begin{array}{llll} u_{x}=u_{y} & =0 & & \;\text{on }\Gamma_{\text{sides}}, \end{array}$$

where u is the displacement vector, h is the initial sample height and $\Gamma_{\text{bottom}}$, $\Gamma_{\text{top}}$, and $\Gamma_{\text{sides}}$ denote the bottom, top, and side surfaces respectively. The prescribed displacement on the top surface was set to 15% of the initial sample height, while the bottom surface was fully constrained. To prevent unrealistic lateral expansion, the nodes on the side surfaces were constrained in the horizontal plane. The quasi-static analysis was performed using 10 time steps. At each time step, the system is solved using the Newton-Raphson method [39,63]:

(B 8)
$$K^{\left(k\right)}\Delta u^{\left(k+1\right)}=-R^{\left(k\right)},$$

where $K^{\left(k\right)}$ is the tangent stiffness matrix, $\Delta u^{\left(k+1\right)}$ is the incremental displacement and $R^{\left(k\right)}$ is the residual vector at iteration k. The solver parameters were set to a maximum of 25 reformations per time step with an optimum of six iterations per step. The simulation output included nodal displacements and element stresses at each time step. Post-processing of the simulation results involved calculating the effective Young’s modulus ($E_{eff}$) of the tissue sample. This was done by analysing the stress-strain relationship:

(B 9)
$$E_{eff}=\frac{\Delta \sigma }{\Delta \epsilon }=\frac{\sigma_{end}-\sigma_{start}}{\epsilon_{end}-\epsilon_{start}},$$

where σ is the average axial stress on the top surface, and ϵ is the applied strain. Within the studied strain range (0–15%), the stress-strain relationship proved to be highly linear ($R^{2}>0.99$), consistent with previous findings on meniscal tissue behaviour under small strains [38]. The normalized (relative) Young’s modulus ($E_{n}$) was then computed as the ratio of the effective modulus to the solid modulus (non-porous):

(B 10)
$$E_{n}=\frac{E_{eff}}{E_{solid}}.$$

This normalized modulus serves as a measure of how the complex microstructure of the tissue affects its bulk mechanical properties.

## Appendix C. Regression analysis methods

To investigate relationships between structural and physical properties, empirical correlations were developed through regression analysis of the 1500 generated samples. These correlations focused on connecting porosity with both hydraulic permeability and mechanical properties. The equation forms were selected after examining various potential relationships, with final selections based on goodness of fit in terms of determination coefficient ($R^{2}$).

To examine the capability of extending these correlations to larger-scale tissue analysis, we suggest a procedure to map CT numbers to porosity values. First, the vascular region is segmented based on its higher relative intensity in the CT image. For an image window W of size 20×20 pixels centred at position (i,j), we compute a local threshold $T_{i,j}$ [64]:

(C 1)
$$T_{i,j}=\mu_{W}+k\sigma_{W},$$

where $\mu_{W}$ and $\sigma_{W}$ are, respectively, the local mean and standard deviation of intensities in window W, and k is a sensitivity parameter set to 1.5. The suitable size of the thresholding window and the standard deviation are set manually to ensure a relatively accurate segmentation. The binary mask M for the vascular region is then:

(C 2)
$$\begin{array}{r} M_{i,j}=\left\{ \begin{array}{ll} 1 & \text{if }I_{CT}(i,j)>T_{i,j}\\ 0 & \text{otherwise}. \end{array}\right. \end{array}$$

The segmented vascular region was then removed through coherent in-painting [56]. For the remaining tissue regions, we established a mapping between normalized CT numbers and porosity. Given that porosity physically ranges from zero (completely solid) to 1 (completely void), we normalize the CT numbers ($I_{CT}$) inversely to porosity (ϕ) using:

(C3)
$$\phi =1-\frac{I_{CT}-min(I_{CT})}{max(I_{CT})-min(I_{CT})}.$$

This mapping ensures that higher CT numbers (indicating denser tissue) correspond to lower porosity values, while maintaining the theoretical bounds of porosity between zero and 1. The inverse relationship between CT intensity and porosity is supported by similar approaches in bone density studies [46,47], where higher CT numbers correlate with greater tissue density and thus lower porosity. While this relationship requires further validation for meniscal tissue, it enables demonstration of how our porosity-based correlations could be applied to analyse property distributions across whole-tissue scales.
